# NLRP3 activation maintains intestinal epithelial barrier and reduces liver injury in alcoholic liver disease mice

**DOI:** 10.1002/ctm2.70099

**Published:** 2024-11-28

**Authors:** Shi‐Qing Li, Ya‐Ru Wang, Zhong‐Liang Xie, Yan Wang, Zi‐Han Feng, Jian‐Hao Xu, Bing Yuan, Yi‐Tong Zhang, Guan Yang, Jing‐Lin Wang, Yuan Yuan

**Affiliations:** ^1^ State Key Laboratory of Pathogen and Biosecurity Academy of Military Medical Sciences (AMMS) Beijing China; ^2^ State Key Laboratory of Respiratory Disease The First Affiliated Hospital of Guangzhou Medical University Guangzhou China; ^3^ Hainan Medical Products Administration Hainan Center for Drug Inspection Haikou China; ^4^ State Key Laboratory of Proteomics Beijing Proteome Research Center National Center for Protein Sciences (Beijing) Beijing Institute of Lifeomics Beijing China; ^5^ Department of Disease Control and Prevention The No. 96609 Hospital of Chinese People's Liberation Army Yinchuan Ningxia China

**Keywords:** alcoholic liver disease, intestinal epithelial barrier, NLRP3 inflammasome, *Vibrio* infection

## Abstract

**Background:**

Alcoholic liver disease (ALD) patients with bacterial infections usually exhibit high mortality rates. Infections frequently involve bacteria such as *Vibrio vulnificus* and *Enterococcus faecalis*. Nevertheless, the mechanisms predisposing ALD patients to bacterial infections and the role of the NLRP3 inflammasome in the intestinal epithelial barrier in ALD remain unclear.

**Methods:**

We established ALD mice models of WT, *Nlrp3* ^−/−^ and *Gsdmd* ^−/−^ through chronic alcohol consumption feeding and acute alcohol induction. We compared alterations in gut microbiota, ileitis, and adhesion protein expression, to analyze the role and potential mechanism of NLRP3 in the early onset of ALD. Concurrently, we examined the changes in inflammation and liver damage in the ileum of ALD and healthy mice following foodborne infection with *V. vulnificus*.

**Results:**

Compared with the control group, the expression levels of ZO‐1, Claudin‐1 and E‐cadherin were reduced in the ileum of ALD mice, while those of NLRP3, caspase‐1(p20), GSDMD‐N and IL‐1β were elevated. *Nlrp3* ^−/−^ and *Gsdmd* ^−/−^ ALD mice showed an increased gut bacterial load, decreased ileal expression of E‐cadherin, more severe ileitis, pronounced liver damage, steatosis and higher plasma levels of FITC‐dextran, D‐LA and ZO‐1 compared with WT mice. Notably, *Nlrp3* ^−/−^ ALD mice exhibited a higher presence of Deferribacterota and Enterobacteriaceae. Furthermore, ALD mice infected with *V. vulnificus* infection exhibited no further activation of NLRP3 in the ileum, leading to increased intestinal permeability and bloodstream infections.

**Conclusions:**

This study indicated that NLRP3 activation in the ileum of ALD mice stabilizes the inflammation‐related gut microbiota, preserves the intestinal epithelial barrier, and diminishes inflammation and liver injury. Furthermore, the compromised immune defence in ALD mice may contribute to their heightened susceptibility to bacterial pathogens.

**Key points:**

- Activation of the NLRP3‐GSDMD pathway in the ileum of Alcoholic liver disease (ALD) mice.
- NLRP3 activation maintains homeostasis of gut microbiota and intestinal epithelial barrier in ALD mice.
- ALD mice infected with *V. vulnificus* infection exhibited no further activation of NLRP3 in the ileum, leading to increased intestinal permeability and bloodstream infections.

## Graphical abstract

NLRP3 activation maintains homeostasis of gut microbiota and intestinal epithelial barrier and reduces liver injury in alcoholic liver disease mice. Meanwhile, after oral infection with *V. vulnificus*, ALD mice did not induce further activation of NLRP3 inflammasomes, leading to disruption of the intestinal epithelial barrier and eventual bloodstream infection.

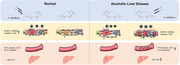

## 1 INTRODUCTION

Alcoholic liver disease (ALD) is a manifestation of alcohol overconsumption and encompasses conditions ranging from alcoholic fatty liver to alcoholic hepatitis, liver fibrosis, and even cirrhosis.^1^ ALD represents a primary cause of chronic liver disease globally, contributing to as much as 48% of cirrhosis‐related mortality in the United States. Moreover, bacterial infections, with a prevalence rate of approximately 20–36%,^2^, ^3^ are frequently observed in ALD patients and are associated with poor outcomes. Infections impact the survival rates of patients at all stages of liver disease.^4^

*Vibrio* spp. is naturally found in marine and estuarine environments, and most illnesses resulting from *Vibrio* infection involve *Vibrio vulnificus, V. cholerae* (non‐O1, non‐O139) and *V. parahaemolyticus*. These bacteria predominantly cause clinical gastroenteritis, wound infections, and septicemia. Infections typically arise following exposure to contaminated seawater or freshwater, or from the consumption of raw seafood.^5^, ^6^ Patients with liver cirrhosis, particularly those with ALD, are highly susceptible to these three types of *Vibrio* bacteria, accounting for over 50% of the infections.^5^, ^6^, ^7^, ^8^ Moreover, these patients face an elevated risk of sepsis and increased mortality.^8^ In addition, patients with ALD who are vulnerable to *V. vulnificus* show varied drinking patterns, ranging from chronic drinking to acute alcoholism, and often become infected after consuming contaminated seafood, especially oysters.^9^, ^10^, ^11^

The gut, as the primary entry point for food‐borne infections, plays a pivotal role in bacterial infections. The migration of bacteria from the gut to the bloodstream constitutes a critical phase in the development of sepsis induced by food‐borne infections. Both chronic alcohol consumption and bacterial infection can disrupt gut permeability, leading to the alteration or reduction of junctions. Consequently, microbial antigens, microbes, and metabolites may translocate to the liver and enter systemic circulation, potentially resulting in endotoxemia or bacteremia.^12^, ^13^

Recent literature has highlighted the role of intestinal inflammation, homeostasis, and other factors in maintaining intestinal permeability.^14^, ^15^ In the context of inflammation, the NLRP3‐mediated pyroptosis pathway is crucial for innate immune function. The NLRP3 inflammasome, a cytoplasmic multiprotein complex, activates caspase‐1 leading to the production of cytokines interleukin (IL)‐1β and IL‐18 and initiates pyroptosis.^16^ Furthermore, the NLRP3 inflammasome interacts with other components involved in apoptosis and necrosis, contributing to a cell death process called PANoptosis, which includes pyroptosis, apoptosis, and necroptosis.^17^, ^18^ Recent studies have suggested the involvement of necroptosis and apoptosis, mediated by molecules such as MLKL and caspase 3/6/7/8 in maintaining intestinal permeability.^19^, ^20^ However, the role of the NLRP3‐mediated pyroptosis pathway in this process remains underexplored. Only an early report mentioned the necessity of the NLRP3 inflammasome for preserving epithelial integrity in experimental colitis,^21^ yet its involvement in maintaining the intestinal epithelial barrier in ALD has not been addressed. Moreover, clinical ALD patients are prone to infections with pathogens such as *V. vulnificus*, *V. parahaemolyticus*, *V. cholerae*, *Escherichia coli*, *Enterococcus faecalis*, and *Candida*.^22^, ^23^ However, the susceptibility mechanism related to the NLRP3 inflammasome remains to be elucidated.

In this study, we established an ALD mouse model susceptible to *V. vulnificus* induced by acute alcoholism based on chronic drinking consumption. Our findings suggest that NLRP3 activation plays a crucial role in maintaining the intestinal epithelial barrier. This is achieved by stabilizing inflammation‐related gut microbiota, enhancing the expression of E‐cadherin and ZO‐1, and reducing intestinal inflammation and liver damage in ALD mice. Consequently, the activation of the NLRP3 inflammasome may be significant for the upkeep of the intestinal epithelial barrier in ALD patients, preventing bacterial translocation in the gut postinfection, and improving the prognosis of sepsis patients. However, following oral infection with *V. vulnificus*, ALD mice did not exhibit further activation of the NLRP3 inflammasomes. This absence of activation might explain the lack of protective effects, leading to the disruption of the intestinal epithelial barrier and subsequent bloodstream infection. Clinical research has indicated that NOD2 variants associated with impaired mucosal barrier function may be genetic risk factors contributing to mortality and spontaneous bacterial peritonitis in patients with liver disease.^24^ This study revealed that NLRP3 activation is beneficial for ALD hosts in maintaining the intestinal epithelial barrier, potentially offering insights into the treatment and prognosis of sepsis in susceptible populations with liver disease.

## 2 RESULTS

### 2.1 Establishment of ALD mice

**FIGURE 1:**
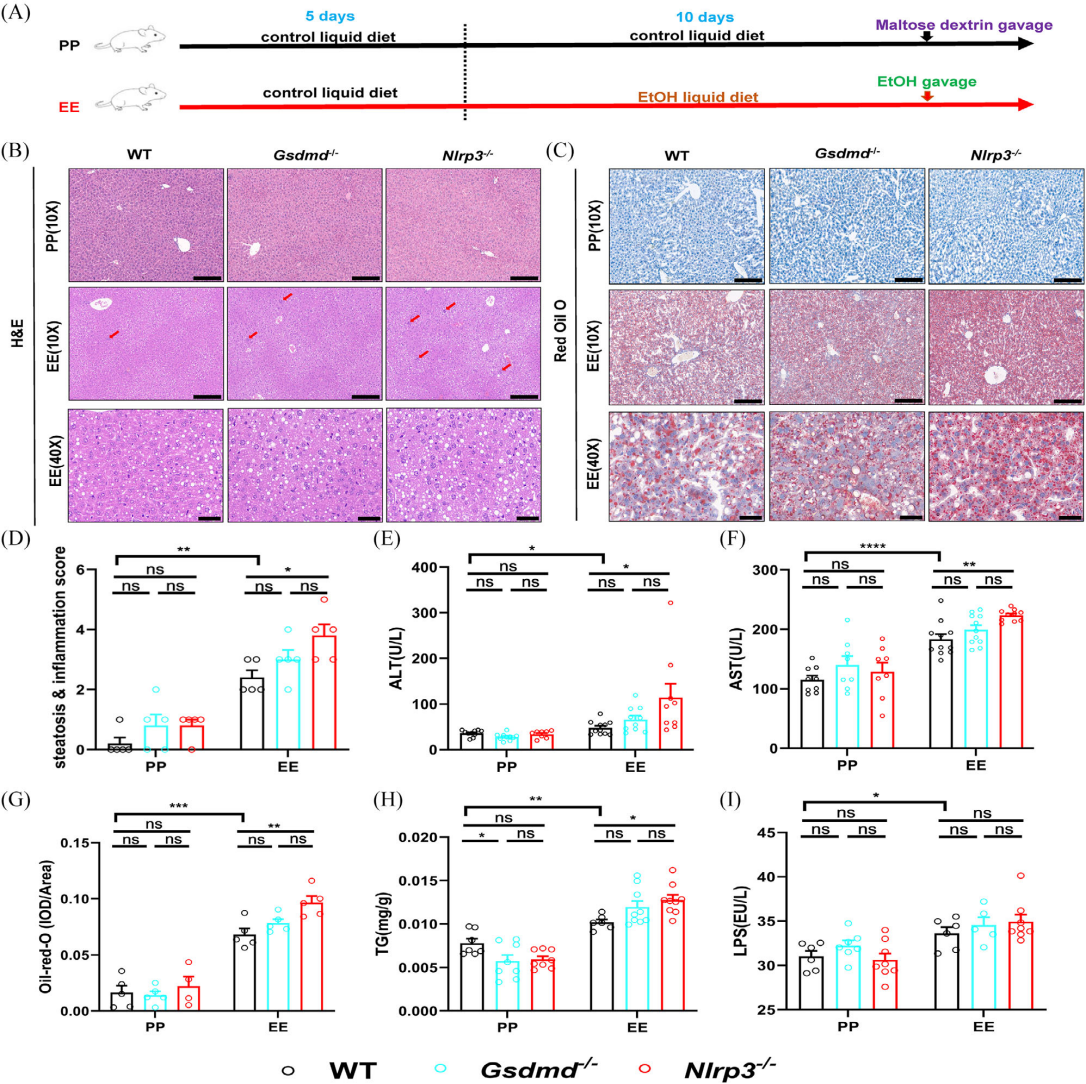
Establishment of a mouse model of alcoholic liver disease (ALD). (A–I) Construction and evaluation of the NIAAA model in mice (two technical replicates). (A) Schematic diagram of the construction of the ALD model. In the NIAAA model, control and alcohol‐fed mice were defined as PP and EE groups, respectively. (B, C) Representative H&E and Oil Red O staining in the liver tissues from PP and EE groups. Scale bars = 200 or 40 µm; red arrows represent inflammatory cell infiltration. (D) Mice liver pathology scored for both hepatocyte inflammation and steatosis (*n* = 5). (E, F) Plasma ALT (E) and AST (F) levels in two groups of wild‐type (WT), *Gsdmd* ^−/−^ and *Nlrp3* ^−/−^mice (*n* = 5–11). (G–I) The data of lipid accumulations were semiquantitatively analyzed as integrated option density (IOD) in Oil Red O staining positive area (*n* = 5). (G) Liver triglycerides (TG) (H) and LPS (I) content were detected (*n* = 6–9). Data are presented as the mean ± standard error of the mean (SEM) (D–I). Statistical analysis was done using one‐way ANOVA in the PP or EE group. *p*‐values detection between PP and EE groups was determined by the unpaired *t*‐test. ns *p* > .05, *p < .05, **p < .01, ***p < .001, ****p < .0001.

To imitate clinical ALD patients and those with bacterial infections, we established ALD mice models according to the NIAAA (National Institute on Alcohol Abuse and Alcoholism) protocol.^25^, ^26^, ^27^ Mice treated with alcohol and those not exposed to alcohol were designated as the EE and PP groups, respectively, as depicted in Figure 1A.

After alcohol feeding and acute alcohol gavage, hematoxylin and eosin (H&E) staining revealed hepatocyte swelling and round vacuoles of varying sizes in the liver tissue of the EE group mice. (Figure 1B). Meanwhile, the hepatocytes in the EE group contained fat aggregated into red lipid droplets stained with Oil Red O and elevated hepatic triglycerides (TG) levels in the liver were tested (Figure 1C,G,H), indicating that feeding mice with alcohol resulted in hepatic steatosis. Moreover, inflammatory damage also be observed in the liver of alcohol‐fed mice (red arrow in Figure 1B), accompanied by elevated liver lipopolysaccharide (LPS) levels (Figure 1I), as well as liver pathology scores (based on hepatic steatosis and inflammation) and elevated plasma ALT and AST levels (Figure 1D–F), suggesting that the NIAAA model feeding led to liver inflammation and injury in ALD mice.

### 2.2 Activation of the ileal NLRP3‐GSDMD pathway in ALD mice

The maintenance of the intestinal epithelial barrier under physiological conditions protects the body from sepsis, which is believed to be associated with cytoskeletal alterations, inflammatory response, intestinal flora disturbance and other mechanisms.^28^, ^29^ However it has not yet been reported whether the NLRP3 inflammasome is involved in maintaining of intestinal epithelial barrier in ALD.

**FIGURE 2:**
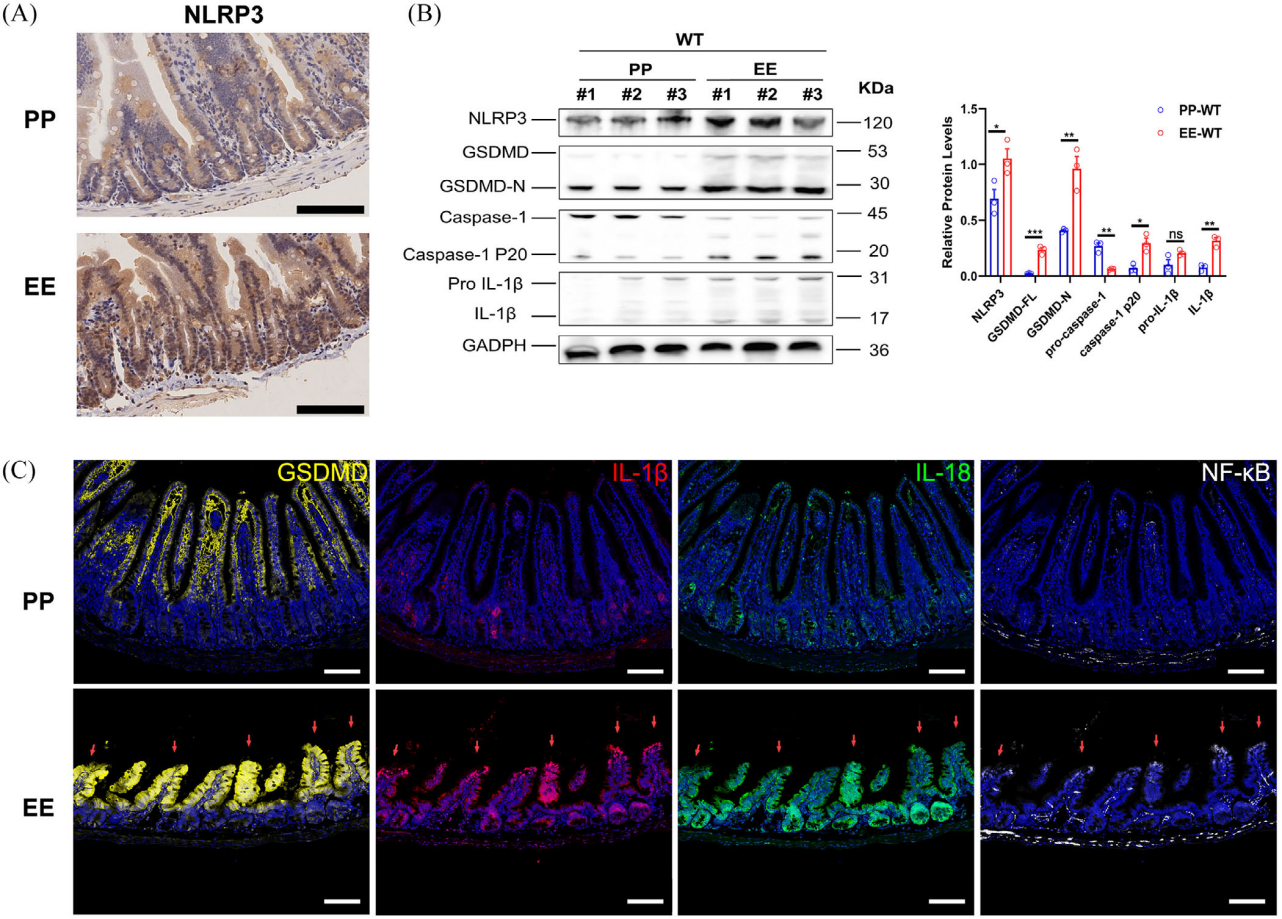
Activation of the Ileal NLRP3‐GSDMD Pathway of WT Mice in EE Group. (A) IHC images of the mouse ileum tissue sections showing localization and expression of NLRP3. Scale bar = 100 µm. (B) The expression of ileal NLRP3‐GSDMD pathway proteins was analyzed by Western blot (*n* = 3). #1, #2, and #3 in both PP‐WT and EE‐WT groups represented different mice respectively. (C) Multiplex IF assay of GSDMD, IL‐1β, IL‐18, and NF‐κB; scale bars = 100 µm. The red arrow indicates a simultaneous increase in protein expression in the ileum. Data are expressed as mean ± SEM (B). *p*‐values were determined by the unpaired *t‐*test. ns *p* > .05, *p < .05, **p < .01, ***p < .001.

Therefore, we performed western blot (WB), immunohistochemistry (IHC), and immunofluorescence (IF) analyses on the ileum of the constructed PP and EE groups of mice to evaluate the expression of the NLRP3‐caspase 1‐GSDMD pathway. Compared with the non‐alcoholic diet, IHC (Figure 2A), WB (Figure 2B), and IF (Figure 2C) indicated that the expression of NLRP3, GSDMD, IL‐1β, IL‐18 and NF‐κB was upregulated in the ileum of EE mice. In addition, activated GSDMD (GSDMD‐N), cleaved‐caspase‐1(caspase p20), and mature IL‐1β levels were increased in the ileum of alcohol‐fed mice (Figure 2B). These results suggested that NLRP3‐caspase 1‐GSDMD signalling is activated in the ileum of alcohol‐fed mice (EE mice).

### 2.3 Alcohol exacerbated gut dysbiosis in ALD mice and *Nlrp3* ^−/−^ mice

**FIGURE 3:**
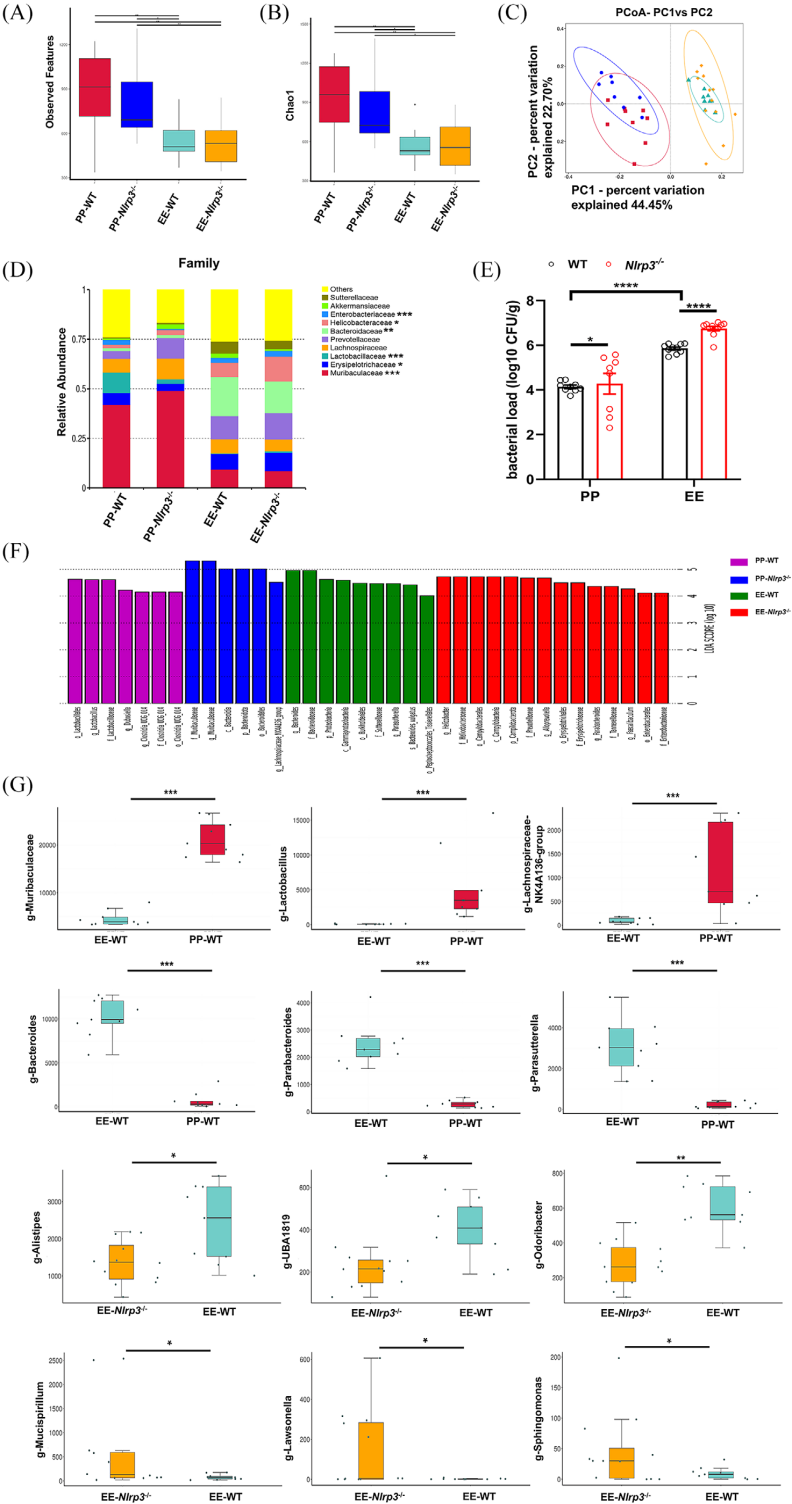
Alcohol exacerbates gut dysbiosis in *Nlrp3* ^−/−^ mice. (A–G) Macrogenomic analysis of faecal microbiota in WT and *Nlrp3* ^−/−^ mice from PP and EE group (PP‐WT *n* = 9, PP‐*Nlrp3* ^−/−^ *n* = 8, EE‐WT *n* = 9, EE‐*Nlrp3* ^−/−^ *n* = 11). Measures of α‐diversity using the total number of observed species (A) and Chao1 index (B). (C) Bray Curtis PCoA weighted Unifrac distances showing a difference in the composition of the faecal microbiota between groups (MRPP test, *p *< .05). (D) Bacterial taxonomic profiling of intestinal bacteria from different groups at the family level. (E) Bacterial count in the ileum of WT and *Nlrp3* ^−/−^ mice in the PP and EE group (PP‐WT *n* = 9, PP‐*Nlrp3* ^−/−^ *n* = 8, EE‐WT *n* = 10, EE‐*Nlrp3* ^−/−^ *n* = 11). CFUs were normalized to grams of intestinal tissue (CFU/g) to represent bacterial counts. (F) Linear discriminant analysis (LDA) scores derived from LEfSe analysis (LDA > 4). (G) Distribution box diagram of bacteria abundance with statistical difference at the genus levels in WT mice from the PP and EE groups and WT and *Nlrp3* ^−/−^ mice from the EE group, respectively, using MetaStat analysis (box plots: median, 25th and 75th percentiles). (A, B) Kruskall–Wallis test followed by the Tukey post hoc test. *p < .05, **p < .01. (D) Statistical analysis was done using one‐way ANOVA compared with PP‐WT or PP‐*Nlrp3* ^−/−^: *p < .05, **p < .01, ***p < .001. (E) Statistical analysis was done using the unpaired *t*‐test. *p < .05, ****p < .0001. Data are shown as the mean ± SEM (E).

Commensal microflora in the lumen of the small intestine plays an important role in maintaining intestinal paracellular permeability.^30^ The relationship between NLRP3 and intestinal homeostasis and permeability in ALD remains unexplored. The *Nlrp3* ^−/−^ ALD mouse model was used and gut microbiota was analyzed. To accurately reflect the influence of gut microbiota on permeability, WT and *Nlrp3* ^−/−^ mice were co‐housed during the model's construction. Faecal samples were subjected to 16S rRNA amplicon sequencing analysis. Alcohol‐fed mice displayed a statistically significant decline in the number, abundance, and diversity of microbial species compared with the control group (PP group) (Figure 3A,B). Beta diversity analysis using a weighted UniFrac distance matrix revealed marked alterations in microbial composition due to ethanol consumption and the absence of NLRP3 (Figure 3C). At the phylum level, specific bacterial taxonomic shifts indicated that alcohol consumption markedly increased the relative abundance of Campilobacterota, Proteobacteria, Desulfobacterota, and Actinobacteria in the faecal microbiota, particularly in *Nlrp3* ^−/−^ mice (Figure S1A). At the family level, Bacteroidaceae, Erysipelotrichaceae, and Sutterellaceae dominated the faecal microbiota in the EE group, whereas Muribaculaceae was prevalent in the PP group. Notably, in alcohol‐fed *Nlrp3* ^−/−^ mice, there was a more significant increase in the relative abundance of Helicobacteriaceae and Enterobacteriaceae in the faecal microflora (Figure 3D). MetaStat tests conducted on WT and *Nlrp3* ^−/−^ mice from both the PP and EE groups revealed significant microbial changes when comparing ALD mice (EE) with normal mice (PP): a reduction in *Lactobacillus, Muribaculaceae* and *Lachnospiraceae* and an increase in *Bacteroides*, *Parabacteroides and Parasutterella*. The relative abundance of *Alistipes* (*Alistipes putredinis* and *A. finegoldii*), *Odoribacter* (*O. splanchnicus*), and *UBA1819* decreased, while that of *Mucispirium* (*M. schaedleri*), *Lawsonella* and *Sphingomonas* increased in the fecal microbiota of *Nlrp3* ^−/−^ mice compared to WT mice in ALD conditions (Figures 3G and S1B). Linear discriminant analysis effect size (LEfSe) also highlighted differences in gut microbiota influenced by alcohol consumption and NLRP3 knockout (Figure 3F). These findings underscore a profound alteration of the gut microbiota under the dual pressures of alcohol and NLRP3 deficiency, accompanied by an increased relative abundance of proinflammation‐related microbiota in *Nlrp3* ^−/−^ mice with ALD, such as Deferribacterota (*Mucispirium*) and Enterobacteriaceae.^30^, ^31^, ^32^ Furthermore, after establishing NIAAA models in both WT and *Nlrp3* ^−/−^ mice, bacterial counts in the ileum were measured. A significantly higher bacteria load was observed in the ileum of *Nlrp3* ^−/−^ mice than in alcohol‐fed WT mice, indicating bacterial overgrowth (Figure 3E).

### 2.4 Ileal inflammation increased in ALD mice and *Nlrp3* ^−/−^ mice

**FIGURE 4:**
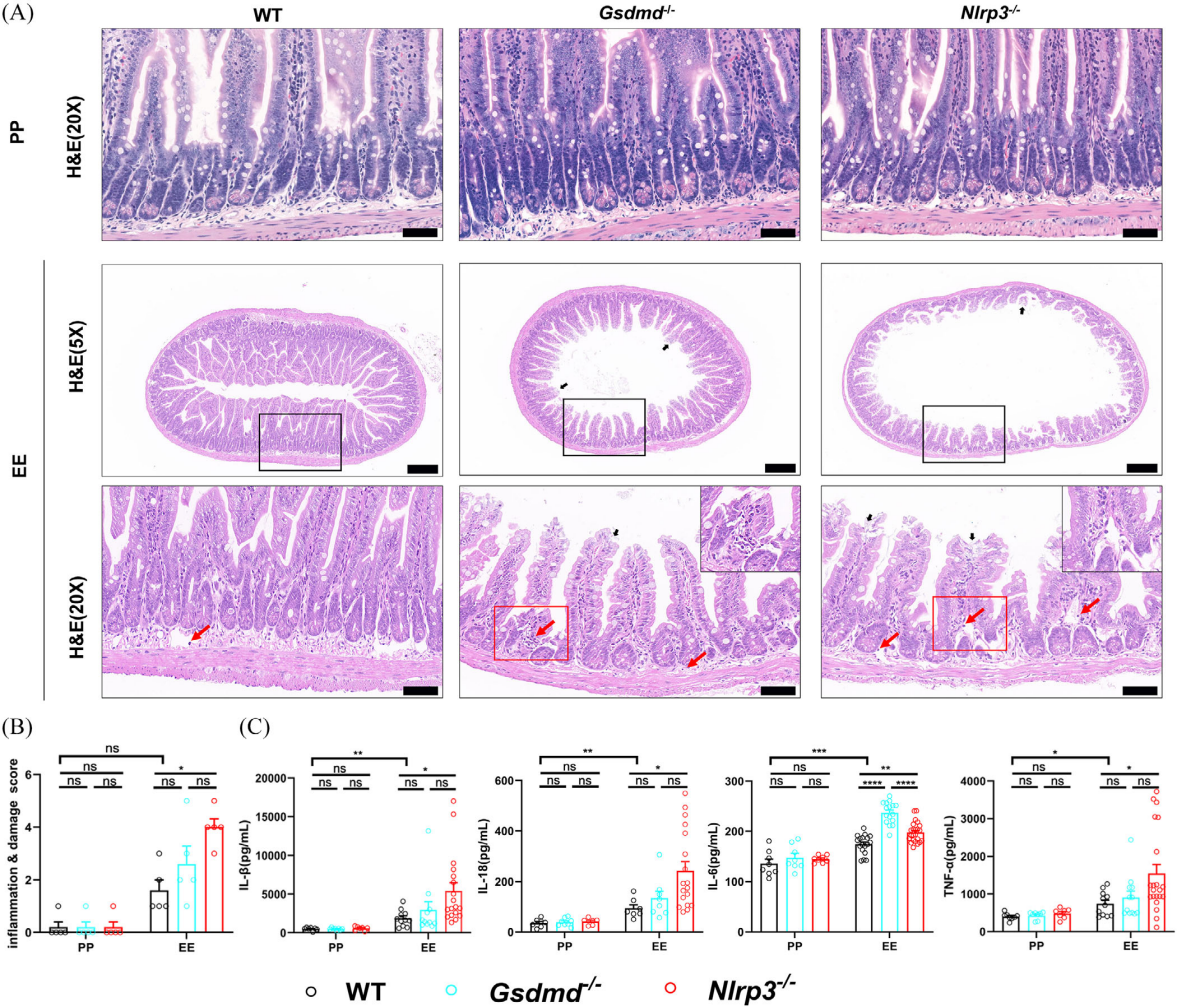
Increased Ileitis in alcohol‐treated *Nlrp3* ^−/−^ mice. (A) Histopathological changes in ileal tissue were examined by H&E staining in PP and EE groups of three types of mice. Scale bar = 200 or 50 µm; black arrows represent villous injury and red arrows represent inflammatory cell infiltration. (B) Pathological score of ileal tissue based on inflammation and villous injury (*n* = 5). (C) Changes in ileal levels of different inflammatory (IL‐1β, IL‐18, IL‐6, and TNF‐α) in PP and EE groups of three types of mice (2–3 technical replicates, *n* = 6–18). Data are presented as mean ± SEM (B and C). Statistical analysis was done using one‐way ANOVA in the PP or EE group. *p*‐values detection between the PP and EE groups was determined by the unpaired *t*‐test. ns *p* > .05, *p < .05, **p < .01, ***p < .001, ****p < .0001.

Numerous studies have indicated that gut dysbiosis is linked to intestinal inflammation, a crucial factor influencing changes in intestinal permeability.^33^ Our experiment also showed that alcohol‐treated mice exhibited increased expression of ileal proinflammatory factors IL‐1β, IL‐18, IL‐6, and TNF‐α (Figure 4C). Interestingly, compared with WT mice treated with alcohol, *Nlrp3* ^−/−^ ALD mice exhibited significantly higher levels of these proinflammatory factors (Figure 4C). Additionally, H&E staining revealed more extensive villous injury (black arrows in Figure 4A) and lymphocyte infiltration (red arrow in Figure 4A) and higher pathological scores (Figure 4B), indicating that alcohol‐treated *Nlrp3* ^−/−^ mice experienced more severe ileitis.

### 2.5 NLRP3 facilitated maintenance of the intestinal epithelial barrier in ALD mice

**FIGURE 5:**
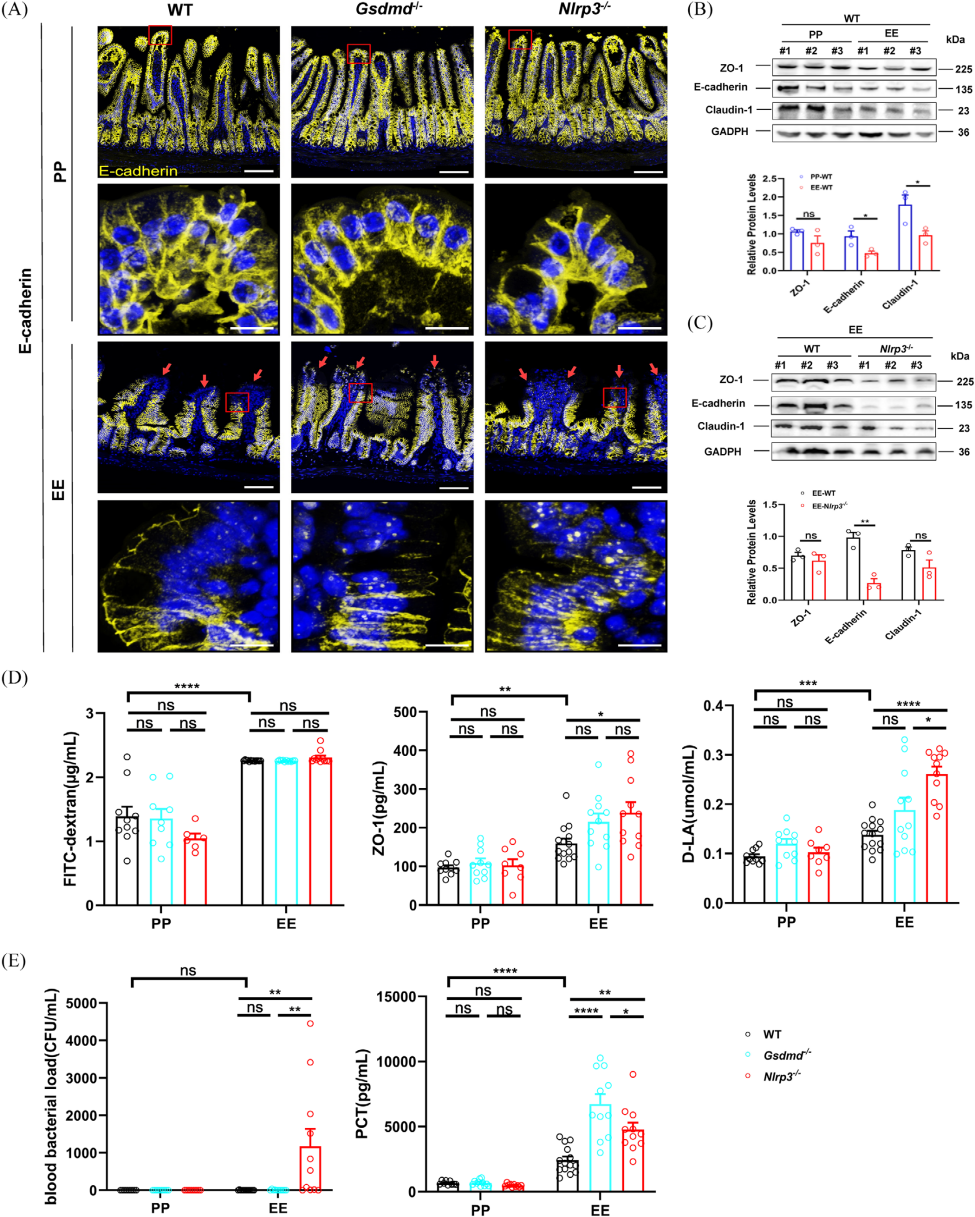
NLRP3 facilitates the maintenance of the intestinal epithelial barrier in the ALD mouse model. (A) Evaluation of E‐cadherin expression and localization in mouse ileum using multiple immunofluorescence based on tyramine signal amplification (TSA) technology. The above pictures were imaged using a BC43 confocal microscope, and the following images were captured using an ultra‐high resolution confocal laser scanning microscopy platform Zeiss LSM880. Scale bar = 5 or 10 µm. The red arrow indicated a decrease in E‐cadherin protein expression at the top of the ileal villi. (B) Western blot display and densitometric analyses of tight junctions (ZO‐1, Claudin‐1) and adherens junctions (E‐cadherin) proteins in PP and EE groups of WT mice (*n* = 3). #1, #2, and #3 in both PP‐WT and EE‐WT groups represented different mice respectively. (C) Western blot display and densitometric analyses of ZO‐1, Claudin‐1 and E‐cadherin proteins in EE groups of WT and *Nlrp3* ^−/−^ mice (*n* = 3). #1, #2, and #3 in both EE‐WT and EE‐*Nlrp3* ^−/−^ groups represented different mice, respectively. (D) Plasma FITC‐dextran, ZO‐1 and D‐LA were measured by ELISA (2–3 technical replicates). (E) Blood bacteria were counted on LBS plates and plasma PCT levels were detected in the PP and EE groups of these three types of mice (*n* = 8–14). Data are presented as mean ± SEM (B–E). Statistical analysis was done using one‐way ANOVA in PP or EE groups. *p*‐values detection between PP and EE groups were determined by the unpaired *t*‐test. ns *p* > .05, *p < .05, **p < .01, ***p < .001, ****p < .0001.

Crosstalk between the intestinal mechanical barrier, gut microbiota, and inflammation jointly affects intestinal permeability. Consistent with other studies,^34^ our results suggest dysregulated junctional proteins (ZO‐1, Claudin‐1, and E‐cadherin) and disruption of intestinal permeability in ALD mice (Figure 5A,B,D). Subsequently, we examined the relationship between the NLRP3‐GSDMD pathway and connexin expression in ALD mice. The intestinal epithelial barrier in *Nlrp3* ^−/−^ and *Gsdmd* ^−/−^ mice was comparable to that of WT mice in the PP group. There was no significant difference in ileal E‐cadherin expression, ileum tissue structure (Figure 5A), and intestinal permeability in the PP group (Figure 5D). However, in the EE group, E‐cadherin expression in the ileal decreased compared with the PP group (red arrow in Figure 5A). Moreover, ultra‐high resolution confocal laser scanning microscopy revealed that E‐cadherin fluorescence lines were more disrupted and punctate in *Gsdmd* ^−/−^ and *Nlrp3* ^−/−^ mice than WT mice in the EE group (Figure 5A). These findings suggest that epithelial adherens junctions were compromised in these mice mediated by E‐cadherin. Correspondingly, H&E staining indicated more severe damage to the intestinal mucosa in *Gsdmd* ^−/−^ and *Nlrp3* ^−/−^ mice in the EE group (Figure 4A). WB analysis also demonstrated decreased expression of ZO‐1, E‐cadherin, and Claudin‐1 in the ileum of *Nlrp3* ^−/−^ mice compared with WT mice in the EE group (Figure 5C). Concurrently, H&E staining showed damaged villi and lymphocyte infiltration in alcohol‐treated *Gsdmd* ^−/−^ and *Nlrp3* ^−/−^ mice (black and red arrows in Figure 4A, respectively). Notably, *Nlrp3* ^−/−^ mice displayed a significant enlargement of the intestinal lumen, damage to the intestinal mucosa and shortening of intestinal villi (Figure 4A). In addition, the plasma level of FITC‐dextran mildly increases in *Nlrp3* ^−/−^ mice relative to WT mice in the EE group (Figure 5D). Both *Gsdmd* ^−/−^ and *Nlrp3* ^−/−^ mice in the EE group exhibited elevated plasma levels of ZO‐1 and D‐LA in their plasma compared to WT mice, with *Nlrp3^−/−^* mice showing particularly higher levels (Figure 5D). Moreover, *Nlrp3^−/−^* mice presented more severe ileal damage and increased plasma levels of D‐LA and ZO‐1 compared to *Gsdmd^−/−^* mice in the EE group. This suggests that the NLRP3 inflammasome, as an important cross‐talk molecule in several death modes, plays a more vital role in maintaining intestinal integrity in ALD than GSDMD, which primarily participates in proptosis. Furthermore, blood bacterial count and procalcitonin (PCT) content were notably higher in *Nlrp3* ^−/−^ mice (Figure 5E). These results suggest that gut barrier disruption is more severe in alcohol‐exposed *Nlrp3* ^−/−^ mice and NLRP3 aids in upholding the intestinal epithelial barrier in ALD mice.

### 2.6 NLRP3 protected against liver injury in ALD mice

Increasing evidence suggests that leaky gut is associated with liver injury.^35^, ^36^, ^37^ Consequently, we investigated the role of the NLRP3 and GSDMD in liver injury in ALD mice. As illustrated in Figure 1E,F, *Nlrp3* ^−/−^ and *Gsdmd* ^−/−^ mice in the EE group had higher AST and ALT activities than WT mice in the same group, especially in *Nlrp3^−/−^* mice. Correspondingly, *Nlrp3* ^−/−^ mice also demonstrated higher scores for steatosis and inflammatory responses in liver tissue compared to WT mice (Figure 1D).^38^ H&E staining and ELISA for liver LPS revealed that *Nlrp*3^−/−^ mice had increased hepatocyte steatosis, lymphocyte infiltration and liver LPS levels relative to WT mice. (Figure 1B,I). Concurrently, Oil Red O staining and liver TG measurement showed that *Nlrp3* ^−/−^ mice possessed more red lipid droplets and higher lipid expression and liver TG content than WT mice (Figure 1C,G,H). Moreover, following acute alcohol consumption, survival rates of *Nlrp3* ^−/−^ mice during subsequent alcohol feeding for 5 days were lower compared with WT mice (Figure S2). These results illustrate that *Nlrp3* ^−/−^ mice fed alcohol exhibit more severe liver damage and reduced survival rates, highlighting that NLRP3 is essential for maintaining normal liver function and improving survival in ALD mice.

### 2.7 Bloodstream infection resulted from gavage infection with *V. vulnificus* in ALD mice

**FIGURE 6:**
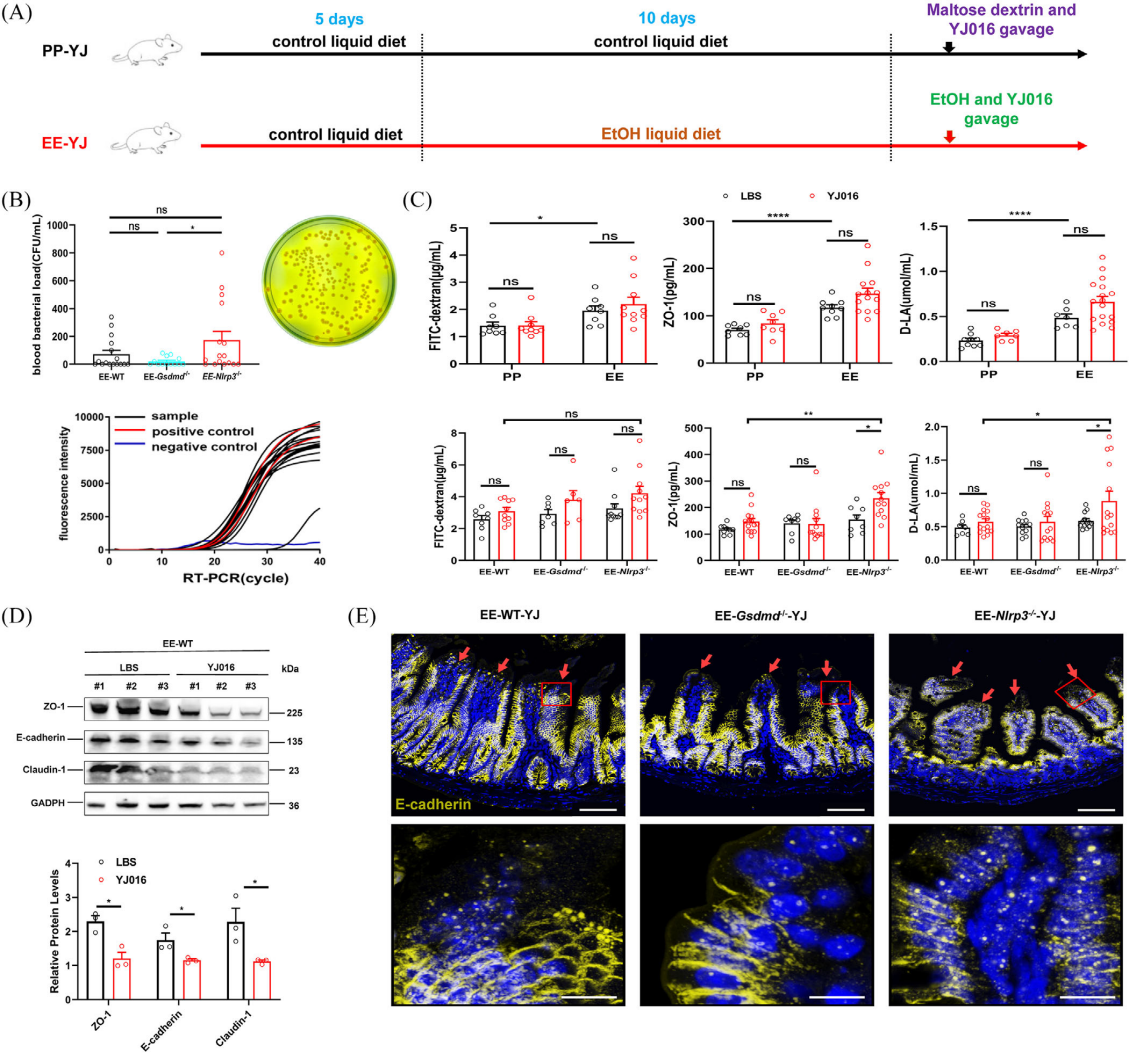
Bloodstream infection caused by *V. vulnificus* YJ016 gavage infection in ALD mice. (A) Schematic diagram of foodborne infection of *V. vulnificus* YJ016 in healthy and ALD mice (2–3 technical replicates). (B) Blood bacterial counting and identification in the EE‐YJ group of WT, *Gsdmd* ^−/−^ and *Nlrp3* ^−/−^ mice (*n* = 7–16). (C) The plasma FITC‐dextran, ZO‐1, and D‐LA levels before and after foodborne infection with *V. vulnificus* YJ016 in healthy and ALD mice constructed by NIAAA model (*n* = 7–17). Meanwhile, plasma FITC‐dextran, ZO‐1 and D‐LA were measured by ELISA in the EE‐YJ group of WT, *Gsdmd* ^−/−^ and *Nlrp3* ^−/−^ mice. (D) Changes in ileal paracellular permeability in EE‐WT groups infected with YJ016 (*n* = 3). #1, #2, and #3 in both EE‐WT‐LBS and EE‐WT‐YJ016 groups represented different mice respectively. (E) Evaluation of ileal E‐cadherin protein expression and localization in the EE‐YJ group of WT, *Gsdmd* ^−/−^ and *Nlrp3* ^−/−^ mice using Multiple IF assay. The above pictures were imaged using a BC43 confocal microscope, and the following images were captured using ultra‐high resolution confocal laser scanning microscopy platform Zeiss LSM880. Scale bar = 5 or 10 µm. The red arrow indicated a decrease in E‐cadherin protein expression at the top of the ileal villi. Data are shown as the mean ± SEM (B–D). Statistical analysis was done using one‐way ANOVA in the EE‐YJ016 group. *p*‐values detection between EE‐LBS and EE‐YJ016 groups were determined by unpaired *t*‐test. ns. *p* > .05, *p < .05, **p < .01, ****p < .0001.

Clinically, patients with chronic alcohol consumption are prone to *V. vulnificus* infections following acute alcohol intake and consumption of raw seafood, presenting a high incidence of sepsis and mortality.^12^, ^13^, ^14^ To replicate this scenario, we constructed an NIAAA model using acute gavage long with simultaneous infection with *V. vulnificus* (10^8^–10^9^ CFU) to assess the onset of bacteremia (Figure 6A). As depicted in Figure 6B, colonies of bacteria in the blood on the modified cellobiose‐polymixin B‐colistin (mCPC) plate were round, flat, and yellow with an opaque centre and transparent periphery, characteristic of *V. vulnificus* colonies. RT‐PCR results indicated that the selected clones were all *V. vulnificus*. The presence of *V. vulnificus* in the bloodstream led to infections in WT, *Gsdmd* ^−/−^, and *Nlrp3* ^−/−^ mice in the EE‐YJ group.

**FIGURE 7:**
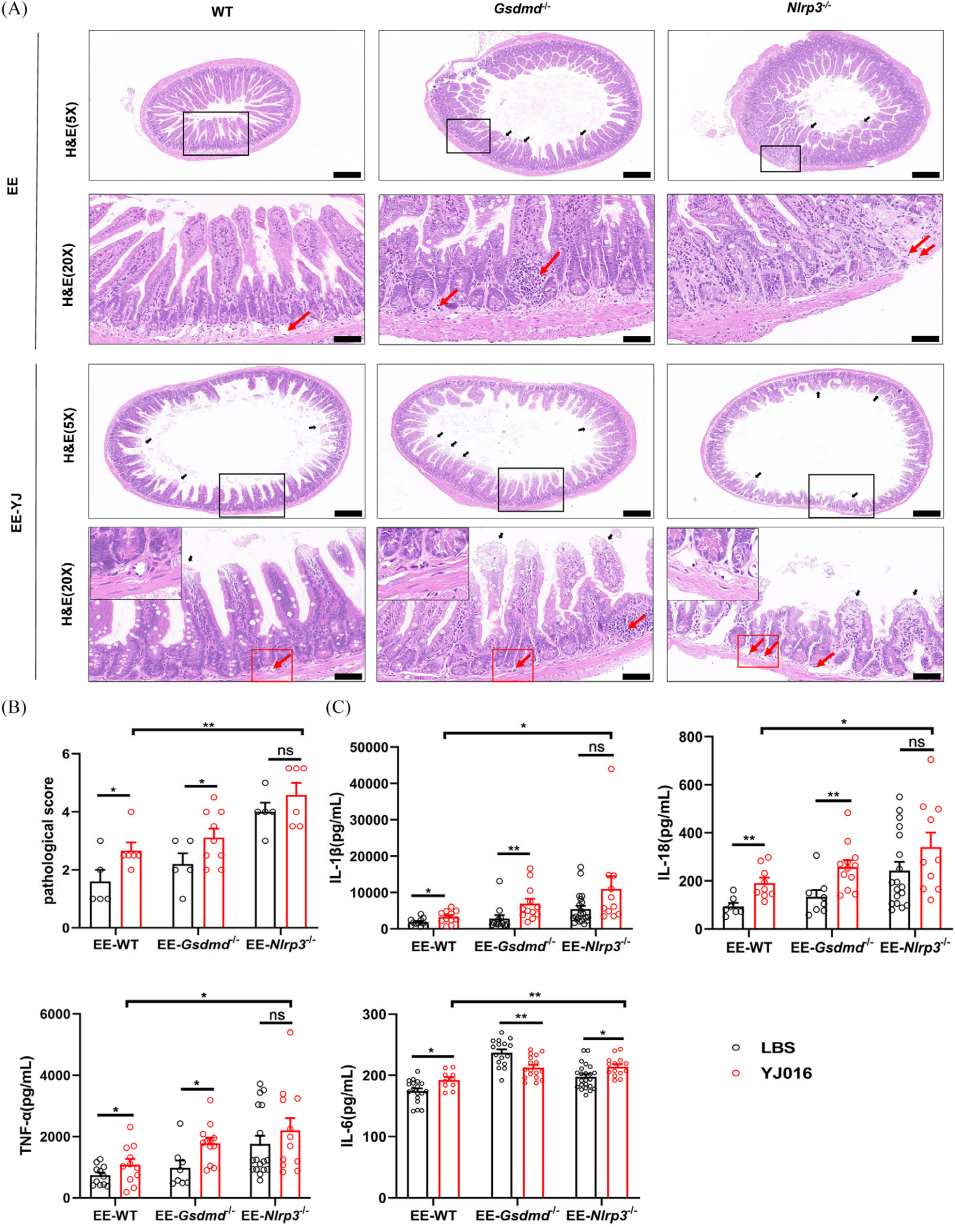
Pathological changes in the ileum caused by *V. vulnificus* YJ016 gavage infection in ALD mice. (A) H&E staining showed pathological changes in the ileum of alcohol‐fed mice infected with YJ016. Scale bars = 200 or 50 µm; black arrows represent villous injury and red arrows represent inflammatory cell infiltration. (B) Ileal pathological score calculated based on villous injury and inflammation (*n* = 5). (C) Changes in ileal levels of different inflammatory (IL‐1β, IL‐18, IL‐6, and TNF‐α) in EE‐YJ group of WT, *Gsdmd* ^−/−^ and *Nlrp3* ^−/−^ mice. Data are shown as the mean ± SEM (B, C). Statistical analysis was done using one‐way ANOVA in the EE‐YJ016 group. *p‐*values detection between EE‐LBS and EE‐YJ016 groups was determined by the unpaired *t‐*test. ns. *p* > .05, *p < .05, **p < .01.

Hence, we assessed changes in the mechanical barrier and permeability of the intestines in both healthy and ALD mice pre‐ and postinfection. Unlike the healthy mice, those treated with alcohol exhibited increased intestinal permeability (Figure 6C), accompanied by a decrease in connexin expression (Figure 6C,E) and disruption of mechanical barriers (indicated by the red arrow in Figure 6E). Furthermore, the fluorescence lines of E‐cadherin appeared more disrupted and punctate in *Nlrp3^−/−^* mice postinfection with *V. vulnificus*, compared to *Gsdmd^−/−^* and WT mice in the EE group (Figure 6E). Consistent with this, H&E staining revealed that the small intestine's mucosal layer in the infected group was thinner, with villus damage (black arrow) and more infiltration of submucosal inflammatory cells (red arrow), particularly notable in *Nlrp3^−/−^* mice (Figure 7A). Further analysis indicated higher levels of FITC‐dextran, D‐LA, ZO‐1, higher blood counts of *V. vulnificus* and more severe ileal inflammation and intestinal villus injury in *Nlrp3* ^−/−^ mice than in WT mice in the EE group infected with YJ016 (Figures 6B,C and 7B,C). These findings suggest that *V. vulnificus* can compromise the intestinal epithelial barrier in ALD mice but not in healthy mice. NLRP3 acts as a crucial innate immune defence molecule, with its activation providing protective effects in ALD mice following infection.

### 2.8 NLRP3 inflammasome was not further activated when ALD mice were infected with *V. vulnificus*

**FIGURE 8:**
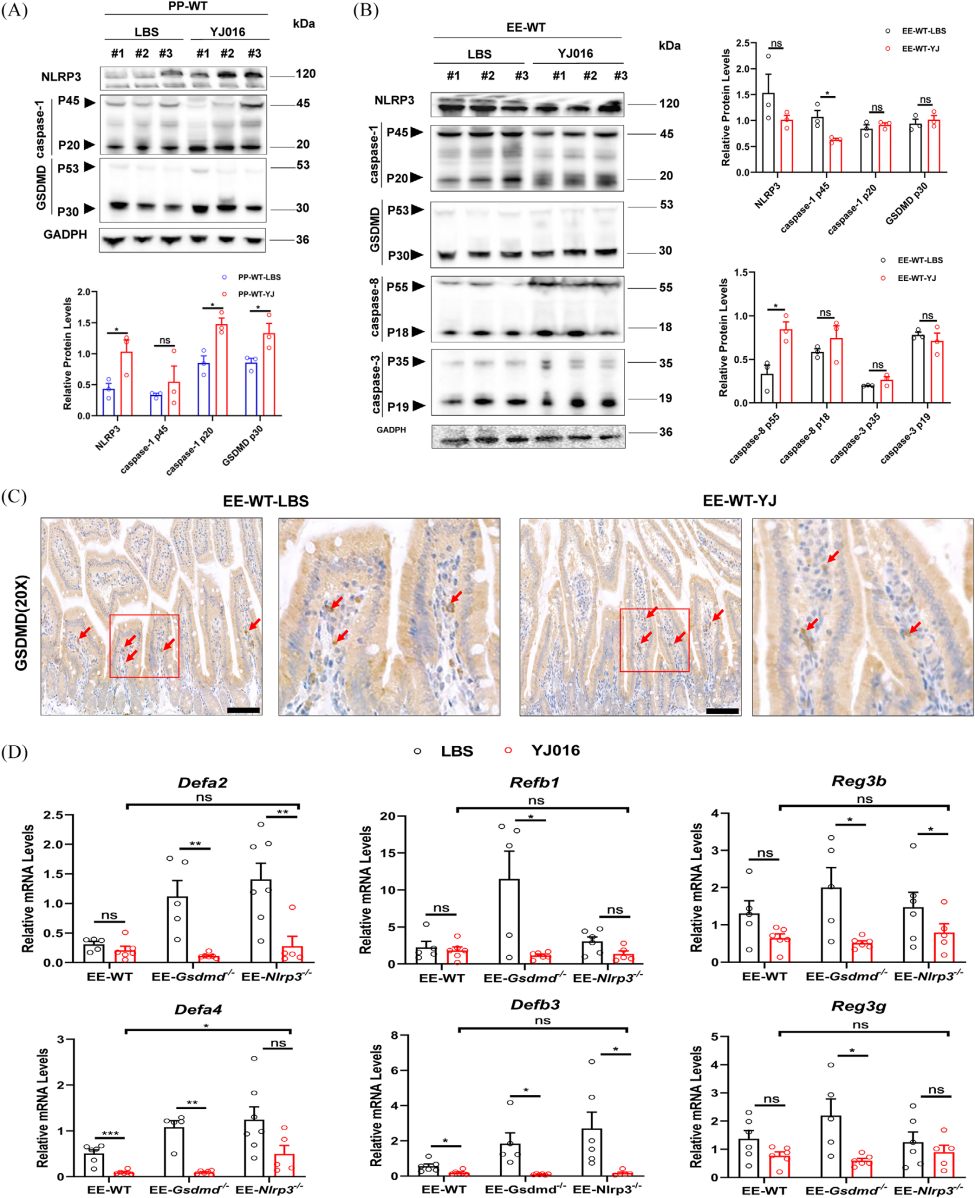
The ileum did not further activate NLRP3 inflammasome when ALD mice were foodborne infected with *V. vulnificus*. (A) Detection of NLRP3‐GSDMD pathway in the ileum of healthy mice infected with *V. vulnificus*. YJ016 by WB (*n* = 3). #1, #2, and #3 in both PP‐WT‐LBS and PP‐WT‐YJ016 groups represented different mice respectively. (B) GSDMD‐mediated pyroptosis and caspase‐3‐mediated apoptosis induced by NLRP3 in the ileum were tested and analyzed in ALD mice infected with YJ016 (*n* = 3). #1, #2, and #3 in both EE‐WT‐LBS and EE‐WT‐YJ016 groups represented different mice respectively. (C) Localization and analysis of GSDMD expression in the ileum of WT mice of ALD infected with YJ016 through IHC; scale bars = 200 µm. (D) The relative mRNA levels of α‐defensin (*Defa1* and *Defa2*), β‐defensin (*Defb1* and *Defb3*), as well as C‐type lectin (*Reg3b* and *Reg3g*) in the ileum of WT, *Gsdmd* ^−/−^, and *Nlrp3* ^−/−^ mice in the alcohol‐treated group before and after infection with YJ016 (n = 5‐7). Data are shown as the mean ± SEM (A, B, and D). Statistical analysis was done using one‐way ANOVA in the EE‐YJ016 group. *p*‐values detection between LBS and YJ016 groups was determined by unpaired *t*‐test. ns. *p* > .05, *p < .05, **p < .01.

Clinical ALD patients, in addition to being susceptible to *Vibrio* infection, may also be infected with bacteria such as *Enterococcus* and *E. coli*, as well as fungi such as *Candida albicans*. However, the infection mechanism has not been extensively reported, and the relationship between this susceptibility and the protective effect of NLRP3 on intestinal permeability remains unclear. Therefore, we investigated the activation of NLRP3 inflammasomes in the ileum following *V. vulnificus* infection. The results showed that, unlike healthy mice which exhibited NLRP3‐caspase‐1‐GSDMD activation, ALD mice did not show NLRP3 inflammasome activation post‐infection. Furthermore, they did not activate the downstream pathway of GSDMD‐mediated pyroptosis and caspase‐3‐mediated apoptosis (Figure 8A–C).

**FIGURE 9:**
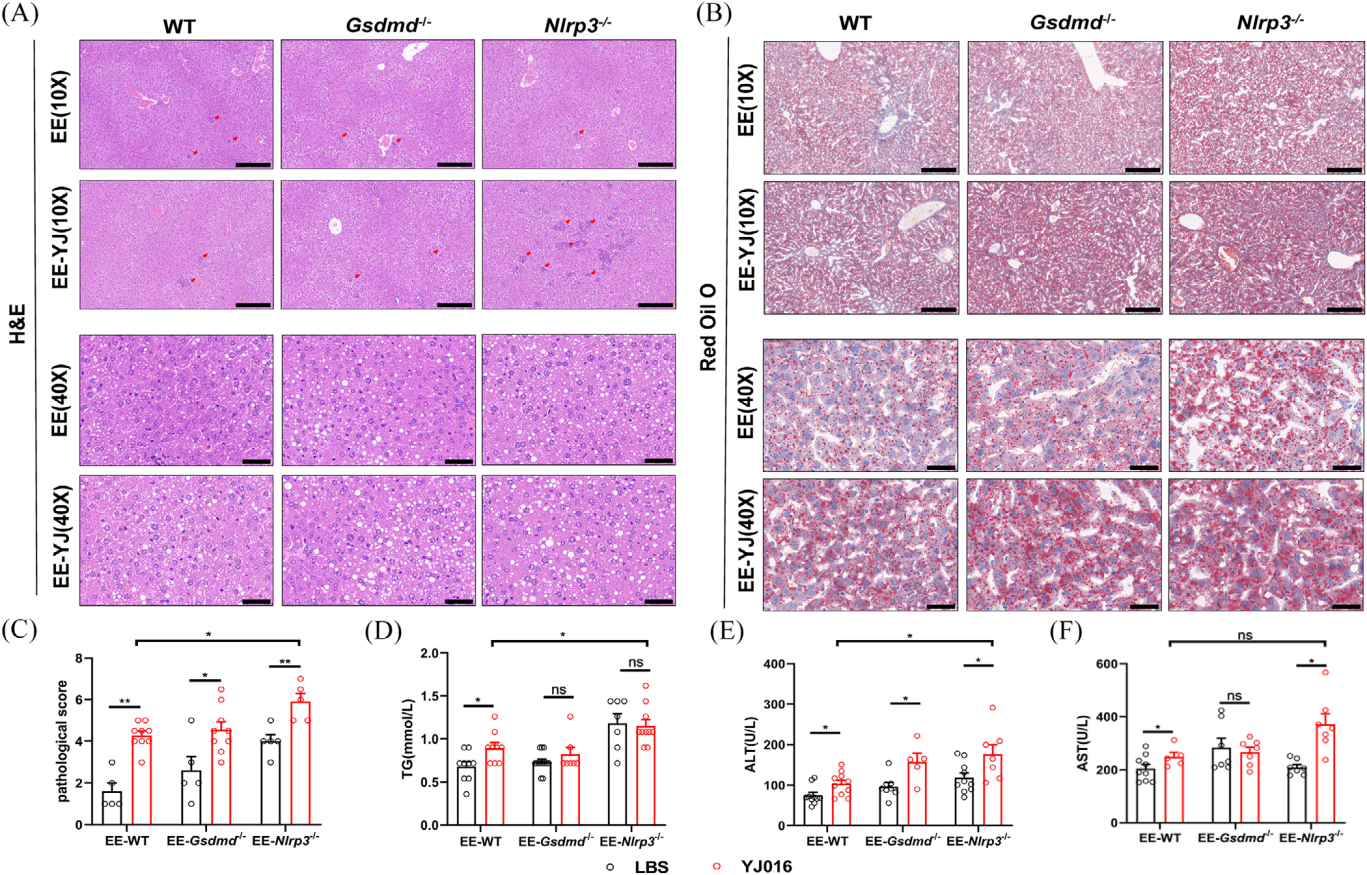
Pathological changes in the liver of ALD mice after foodborne infection with *V. vulnificus*. H&E (A) and Oil Red O (B) staining of the liver before and after infection with *V. vulnificus* in EE group of WT, *Gsdmd* ^−/−^ and *Nlrp3* ^−/−^ mice. Scale bars = 200 or 40 µm; red arrows represent inflammatory cell infiltration. (C) Pathological scoring based on liver steatosis and inflammation (*n* = 5–9). Plasma TG (D), ALT (E), and AST (F) levels before and after infection in ALD mice (*n* = 5–10). Data are shown as the mean ± SEM (C–F). Statistical analysis was done using one‐way ANOVA in the EE‐YJ016 group. *p*‐values detection between LBS and YJ016 groups was determined by unpaired *t*‐test. ns. *p* > .05, *p < .05, **p < .01.

Defensins, known as antibacterial properties, are reported to play a protective role against bacterial infections. In WT, *Gsdmd^−/−^* and *Nlrp3* ^−/−^ ALD mice infected with *V. vulnificus*, the expression levels of α‐defensin (*Defa2*, *Defa4*), β‐defensin (*Defβ1*, *Defa3*), and C‐type lectin (*Reg3b*, *Reg3g*) in the ileum were reduced, suggesting compromised microbial defence in these mice (Figure 8D). This reduction might contribute to their increased vulnerability to pathogens. Furthermore, liver function tests also revealed that, unlike healthy mice, infection with *V. vulnificus* exacerbates liver damage and steatosis in ALD mice, leading to elevated plasma levels of ALT, AST, and TG, as well as higher liver pathological scores in the EE group postinfection (Figures 9; Figure S3). These findings indicate that infection in ALD mice worsens liver damage, and partially explaining the elevated rates of sepsis and mortality observed in patients with liver disease following *V. vulnificus* infection in clinical settings.

## 3 DISCUSSION

Dysfunction of the intestinal barrier is associated with the natural history of cirrhosis and is significantly contributes to increased susceptibility to food‐borne infections.^39^ NLRP3 and GSDMD, crucial components of the innate immune response that lead to pyroptosis, have been reported to be activated in the liver of ALD mice.^40^, ^41^ Moreover, levels of IL‐1β, IL‐18, and caspase‐1 are notably higher in both human and murine livers affected by ALD.^40^, ^42^, ^43^ Nevertheless, the activation of the intestinal NLRP3‐GSDMD pathway in ALD mice remains unexplored, as does the involvement of NLRP3 in maintaining intestinal permeability or its specific mechanism in this context. This study shows that the activation of NLRP3 in the ileum in ALD mice induces caspase‐1 activation of and the cleavage of GSDMD and IL‐1β (Figure 2). It also shows that activation of the NLRP3 inflammasome is essential for maintaining epithelial integrity in ALD mice, evidenced by increased levels of FITC dextran, D‐LA, and ZO‐1 in the plasma of *Nlrp3* ^−/−^mice from the EE and EE‐YJ groups.

The gut microbiota is closely associated with intestinal homeostasis, which influences the inflammatory response, mechanical barrier function, and intestinal permeability.^33^, ^44^ Our findings indicate that NLRP3 activation in ALD mice can regulate gut microbiota composition by affecting the balance of the intestinal flora (Figure 3). Compared with normal mice (PP), WT mice fed with alcohol (EE) exhibited a similar alteration in faecal microbial composition as observed in patients with ALD or cirrhosis.^45^, ^46^, ^47^ Further analysis revealed that a portion of the increased microbiota is associated with proinflammatory responses, such as *Parasutterella*, while another part is related to anti‐inflammatory reactions, like *Parabacteroides* (*P. goldsteinii* and *P. distasonis*). This suggests that in the early stages of ALD, the bacterial community could mitigate alcohol‐induced damage, leading to mild intestinal inflammation and relatively intact mechanical barriers. Remarkably, alcohol‐fed *Nlrp3^−/−^* mice demonstrated a stool microbial composition characterized by a relative overgrowth of potentially pathogenic taxa, Deferribacterota (*Mucispirium*), Enterobacteriaceae, and *Faecalibaculum*, linked to proinflammatory responses and ALD progression.^30^, ^31^, ^32^, ^48^ Additionally, a decrease in *Alistipes* (*A. finegoldii*) and *Odoribacter* (*O. splanchnicus*), which have protective roles against inflammatory bowel disease, was noted in the faecal flora of *Nlrp3^−/−^* mice.^49^, ^50^ Intestinal bacteria were also increased in *Nlrp3^−/−^* mice with ALD. Changes in the types and quantities of gut microbiota collectively led to more severe ileitis and more severe disruption of the intestinal mechanical barrier in alcohol‐fed *Nlrp3^−/−^* mice. These results indicated that early activation of NLRP3 under alcohol feeding leads to microbiota changes associated with inflammation, thereby maintaining intestinal homeostasis and permeability.

In addition to the crucial role of NLRP3 (a key molecule that interferes with different modes of death) by regulating the microbiota in intestinal stability, GSDMD (the effector molecule of pyroptosis) also plays a role in intestinal homeostasis in ALD mice. Compared with WT mice, *Gsdmd* ^−/−^ mice of ALD also showed a certain degree of aggravation of ileitis, villus damage, and damaged intestinal barrier. However, as a downstream of NLRP3, its protective effect is weaker than that of NLRP3. Consistent with NLRP3, GSDMD plays a role in maintaining homeostasis, related to the release of gut microbiota and inflammatory factors.^51^, ^52^ For example, GSDMD maintained the gut barrier of high‐fat diet mice and prevented systemic infections by directly killing Proteobacteria phylum via directly interacting with Cardiolipin.^51^ Besides, GSDMD also maintains intestinal homeostasis by promoting mucus secretion and directly extrusion infected cells.^53^, ^54^, ^55^, ^56^ For instance, the activation of intestinal epithelial GSDMD is related to the secretion of mucus by goblet cells and the remodelling of the host–microbe interface.^54^ All of these indicate the important role of GSDMD mediated inflammation and pyroptosis in maintaining intestinal integrity.

Moreover, excessive alcohol consumption can induce gut leakiness, allowing gut microbes and their byproducts to infiltrate the liver and circulatory system. This incites immune responses, leading to liver damage across various cell types.^12^ However, the role of NLRP3 activation in ALD remains controversial. Some studies have suggested that NLRP3 deficiency exacerbates liver damage and steatosis in mice.^57^, ^58^ Conversely, other studies indicated that NLRP3 deficiency attenuates alcoholic liver steatosis and damage.^43^ These disparate findings may stem from variations in animal sex or experimental conditions. Building on prior research,^57^, ^58^ our study demonstrated that *Nlrp3^−/−^* mice exhibit increased hepatic injury, characterized by elevated plasma ALT and AST levels, along with enhanced liver steatosis and lipid expression. These observations suggest that the NLRP3 inflammasome plays a protective role in mitigating ethanol‐induced hepatic damage and steatosis.

In clinical practice, the majority of *V. vulnificus* infection cases occur in patients with alcohol‐related liver cirrhosis or immunocompromised states; however, it typically does not cause severe illness in healthy individuals.^59^ In the context of alcohol‐related liver disease, patients with ALD and chronic drinking habits who consume raw seafood during episodes of acute alcohol abuse are particularly susceptible to *V. vulnificus*, exhibiting a high incidence of sepsis and mortality.^9^, ^10^, ^11^ To recreate this scenario, YJ016 was administered to mice concurrently with acute alcohol gavage in the NIAAA model. The detection of *V. vulnificus* in the bloodstream eight hours postinfection indicates that infection with *V. vulnificus* in ALD mice can result in bloodstream infection in the NIAAA model. This model offers valuable work for exploring the mechanisms underlying acute infections in individuals with liver disease, which remain incompletely understood.

**FIGURE 10:**
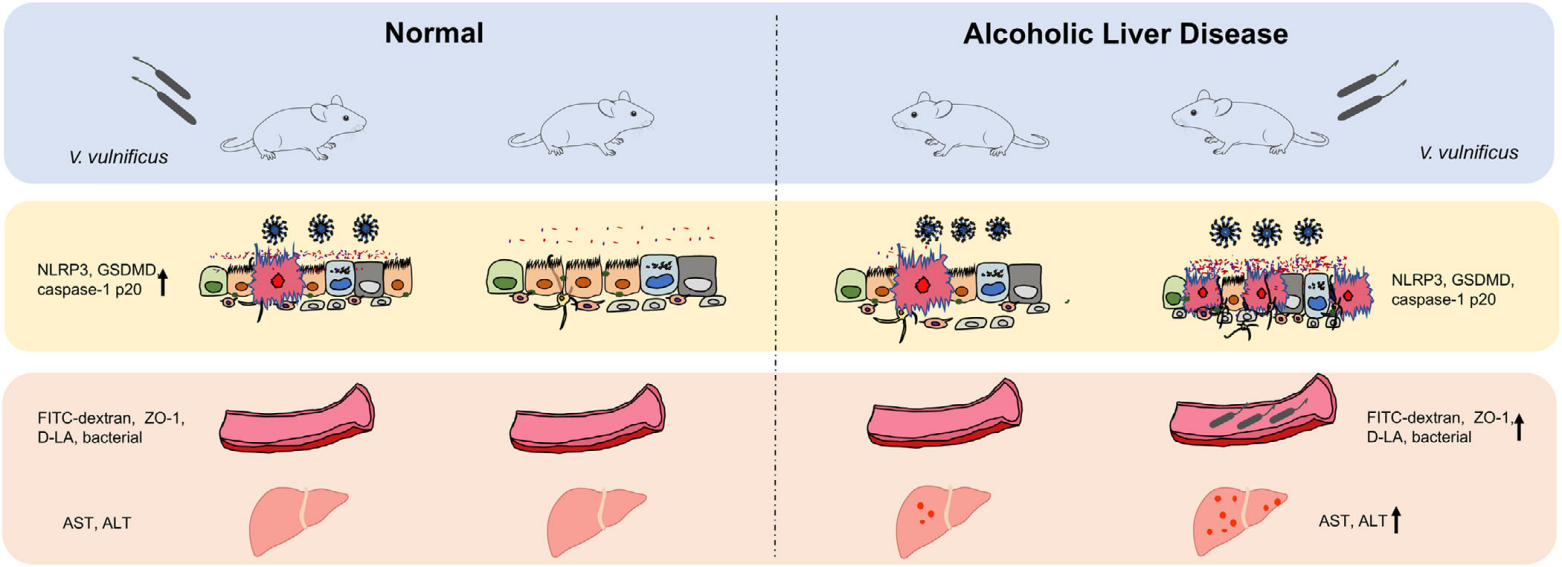
NLRP3 activation maintains homeostasis of gut microbiota and intestinal epithelial barrier and reduces liver injury in alcoholic liver disease mice. First, NLRP3 activation maintains homeostasis of gut microbiota and intestinal epithelial barrier and reduces liver injury in alcoholic liver disease mice. The activation of NLRP3 in the ileum of ALD mice influenced the abundance of inflammation‐related gut microbiota to maintain a balance of intestinal homeostasis, which in turn prevented subsequent intestinal inflammation (IL‐1β, IL‐6, TNF‐α, etc.), increased the expression of connexin (E‐cadherin, ZO‐1), maintained intestinal barrier function, and reduced liver inflammation and steatosis, leading to the reduction of bacteria in the blood. *Nlrp3* ^−/−^ ALD mice exhibited a higher abundance of proinflammatory gut bacteria (Deferribacterota and Enterobacteriaceae), less ileal expression of E‐cadherin and ZO‐1, more severe ileitis, elevated plasma levels of FITC‐dextran, D‐LA, ZO‐1, and more bacteria, as well as more pronounced liver damage and steatosis compared with WT mice. Then, when ALD mice are infected with *V. vulnificus*, due to the activation of its NLRP3, it cannot fully activate NLRP3 after infection, thereby failing to exert its antibacterial protective effect, leading to the occurrence of bloodstream infection.

Previous studies have identified an immune “exhaustion” phenomenon in patients with ALD, characterized by an overactivation of the immune system, that hinders their ability to combat bacterial infections effectively.^60^, ^61^ Our preliminary research revealed that ALD mice infected with *V. vulnificus* showed a significant reduction in the expression of genes associated with bacterial defence compared to healthy mice.^62^ Furthermore, by integrating findings from existing studies and our experimental results, we hypothesize that ALD patients exhibit an immune deficiency state characterized by sustained non‐specific inflammatory responses, which impairs their ability to activate the NLRP3 inflammasome postinfection, thereby compromising their antibacterial defences (Figure 10). Subsequently, this cascade of events leads to bloodstream infections, evidenced by heightened intestinal villus damage, decreased defensin levels, disrupted intestinal mechanical barriers, increased intestinal permeability, and exacerbated liver damage in ALD mice following *V. vulnificus* infection, as depicted in Figures 6, 7, 8, 9. These results suggest that activation of the NLRP3 inflammasome likely initiates beneficial responses in the early phase of ALD. NLRP3 agonists may hold therapeutic value for treating early‐stage ALD and those ALD patients with bacterial infections.

## 4 MATERIALS AND METHODS

### 4.1 Bacterial strains

*V. vulnificus* YJ016, a clinical strain isolated from patients with sepsis, was cultured as previously described.^62^

### 4.2 Ethics statement

Male C57BL/6 mice (9–11 weeks of age, approximately 27–30 g) were purchased from Beijing Vital River Laboratory Animal Technology Co. and used as WT mice. *Nlrp3^−/−^* and *Gsdmd^−/−^* mice in the C57BL/6 mice were purchased from GemPharmatech (T010873, T010437). All animals were housed at 22  ± 4°C under 12 h light/12 h dark cycles before exposure to specific pathogen‐free conditions. Animal study protocols were approved by the Ethics Committee of the Academy of Military Medical Sciences (Ethics Board approval number: IACUC‐DW ZX‐2020‐008).

### 4.3 Experimental infections of mice

The NIAAA model for ALD was used, as shown in Figure 1A. For the NIAAA model,^25^, ^26^, ^27^ mice were fed the Lieber‐DeCarli diet for 15 days. To help mice adapt to a liquid alcohol diet, the alcohol group mice were given a nonalcoholic adaptive diet for 5 days and received a 5% Lieber DeCarli EtOH (volume/volume) alcohol liquid diet (Lieber, Dyets, Inc.) for 6–15 days. On the 16th day, 31.5% (volume/volume) ethanol was administered by gavage, known as the EE group (EtOH feeding + EtOH gavage). In contrast to the EE group, we named the nonalcoholic feeding group the PP. The PP group received a diet with an isocaloric substitution of dextrose for 15 days and was administered 45% (w/v) maltodextrin by gavage on day 16. Mice in the EE group were administered YJ016 by gavage (10^8^∼10^9^ colony forming units; CFU) and 31.5% ethanol together and sacrificed 8 h later. Whole blood (anticoagulated with sodium hydrochloride) was collected by cardiac puncture, and the blood bacterial load was measured using mCPC plates (40 U/mL polymyxin B). The small intestine and liver of mice were collected for subsequent bacterial count, hematoxylin and eosin (H&E) and Oil Red O staining. IHC, IF, and WB analyses were also carried out.

### 4.4 H&E and Oil Red O staining or analysis

Ileum and liver tissue were fixed with 4% paraformaldehyde, embedded in paraffin, cut into 5 µm‐thick sections, and stained with H&E according to the manufacturer's protocols (G1003, Servicebio). Pathologists used a blinded method to score the histology of the ileum and liver. The ileum score was divided into inflammation (score 0–3) and tissue damage (score 0–3).^21^ Liver pathological scores were generated based on hepatocyte steatosis (score 0–4) and inflammation (score 0–4).^38^ For Oil Red O staining, liver samples were embedded in the OCT compound and stained. Lipid accumulation data were analyzed using Image‐Pro Plus software according to integrated option density for the positive area of the microphotograph.^63^

### 4.5 Blood bacterial load and biochemical assays

To measure the number of bacterial CFUs in the blood, 100 µL whole blood and serial dilutions were prepared, followed by plating 100 µL onto Luria–Bertani salt medium (LBS) plates or mCPC plates containing polymyxin B (40 U/mL). The clones on the plates were counted and identified by RT‐PCR. The plasma is obtained by centrifugation from the remaining blood at 3000 rpm for 15 min at 4°C, and those were separated for the determination of ZO‐1, D‐LA, and PCT by ELISA according to kit instructions. Levels of TG, AST and ALT were measured by using an automatic biochemical analyzer (Biobase), the specific product numbers are listed in Table S1.

### 4.6 Small intestine bacterial load and ileal inflammation and liver steatosis and inflammation

The ileal tissue of the mice was washed, collected in 1 mL phosphate‐buffered saline (PBS) buffer, and homogenized by grinding. Different dilutions (100 µL) of the homogenized sample were plated on LBS plates and incubated at 37°C for 24–48 h. The ileal tissue and livers were homogenized and centrifuged, and levels of IL‐1β, IL‐18, TNF‐α, IL‐6, LPS, and TG were determined using assay kits by strictly following the instructions, the specific product numbers are listed in Table S1.

### 4.7 Bacterial DNA extraction and 16S rRNA amplification sequencing

The genome DNA of stool samples from WT and *Nlrp3^−/−^* mice of the PP and EE groups was extracted using a Stool DNA Kit (TianGen, Catalog #: DP712). The faecal DNA was used as the template to amplify the V3‐V4 region of the 16S rRNA genes for subsequent pyrosequencing. The PCR primers, F‐CCTAYGGGRBGCASCAG, R‐GGACTACNNGGGTATCTAAT. The reaction and cycle of PCR are carried out according to the instructions of the kit (Phusion High‐Fidelity PCR Kit, New England Biolabs). Separate and purify the amplified products, and finally collect them on the Illumina NovaSeq platform (Illumina Inc.) for sequencing.

Using FLASH (V1.2.11, http://ccb.jhu.edu/software/FLASH/) and fastp (Version 0.23.1) to obtain clean data. Then, based on clean data, the DADA2 module in QIIME2 software (QIIME2‐202006 version) was used for denoising to obtain the final amplicon sequence variants. The sequence data reported in this study have been deposited in the NCBI Sequence Read Archive (http://www.ncbi.nlm.nih.gov/sra) under the accession number SUB13793496.

### 4.8 Real Time‐PCR (RT‐PCR)

*V. vulnificus* colonies in the blood were identified by their ability to ferment cellobiose on mCPC plates to form yellow colonies. Each colony was identified by RT‐PCR using specific primers and a probe against the *V. vulnificus vvp* (the gene encoding a metalloprotease), as previously described.^64^ For each plate, one of five *V. vulnificus* colonies was analyzed.

Meanwhile, total RNA was extracted from mouse small intestines were isolated using TRIzol reagent, reverse transcribed (TaqMan Reverse Transcription Reagents; Thermo Fisher Scientific) and amplified with SYBR green PCR master mix (Vazyme, Q711‐03). Primers of defensin as described earlier,^65^, ^66^ the specific sequence can be found in Table S2, and the relative expression levels of the different genes were normalized to the GADPH gene and calculated using the 2^^−△△Ct^ method setting the values of PP‐fed as one.

### 4.9 Mice intestinal permeability assay

Intestinal permeability of mice was assessed by oral gavage of 12 mg/mouse 4 kDa FITC‐dextran reconstituted in PBS (46944; Sigma‐Aldrich) 4 h prior to sacrifice. The plasma concentration of FITC‐dextran was tested (Em:485 nm; Ex:535 nm) in duplicate using a fluorescent plate reader (Thermo Scientific Varioskan Flash) and calculated using serial dilutions of FITC‐dextran as standards.

### 4.10 Western blot analysis

After ablation of the liquid nitrogen quick‐frozen intestinal tissue, extraction of the total intestine and mesentery proteins was completed according to the manufacturer's procedure (SA‐05‐IM; Invent Biotechnologies, Inc.) and quantitatively analyzed using the bicinchoninic acid method (PA101‐01; Biomed). Proteins (20–30 µg) were separated on 4–12% or 4–20% polyacrylamide SDS‐PAGE gels (M00656; GenScript) and electro‐transferred to polyvinylidene difluoride membranes (IPVH00010; Millipore). The membrane was locked and incubated for 24 h at 4°C with the primary antibody (1:1000 or 1:1500) and then for 1 h at room temperature with secondary antibodies (1:5000). Finally, blots were visualized with an ECL chemiluminescence reagent (P90719; Millipore) using the ImageQuant LAS4000 system (GE Healthcare). The antibodies used are listed in Table S3. Densitometry reports indicate the mean ± SEM after normalization to the loading control, calculated using ImageJ software.

### 4.11 Immunohistochemistry analysis

Mouse ileal tissue samples were fixed and embedded consistent with H&E staining. Tissues (about 5 µm) were stained with anti‐NLRP3(1:850, ab214185, Abcam) and anti‐GSDMD (1:200, # 39754S, Cell Signaling Technology). The slides were stained with diaminobenzidine, and images of mouse ileum tissue samples were pan‐scanned using a 3DHISTECH instrument (Pannoramic 250 FLASH, H‐1141 Budapest, Öv u. 3.).

### 4.12 Multiplex immunofluorescence assay

Multiple IF (Multiplex IF) analysis based on tyramine signal amplification (TSA)‐fluorophores (Histova Biotechnology, NECC7100) technology was performed on 4–6 µm paraffin sections. Briefly, continuously incubating the antigens in the slices with different first antibodies for 2 h, and then labelling them with HRP conjugated second antibodies and corresponding TSA fluorescent groups. The slides in the recovery/elution buffer (Histova Biotechnology, ABCFR5L) were heated using the microwave at 95°C for 10 s to eliminate primary and secondary antibodies between different antigen and TSA fluorophore combinations. Finally, slides were imaged using BC43 confocal microscope (Oxford Instruments) or the ultra‐high resolution confocal laser scanning microscopy platform Zeiss LSM880 equipped with 405‐nm/488‐nm/543‐nm/594‐nm/633‐nm lasers. The antibody of E‐cadherin (1:400, #3195), and NF‐κB (1:400, #8242s) were from Cell Signaling Technology. The antibody of GSDMD (1:1000, ab219800), IL‐1β (1:400, ab283818), and IL‐18 (1:400, ab223293) were from Abcam. Data were further processed and statistically analyzed using the ZEN and Bitplane Imaris software (Bitplane AG).

### 4.13 Statistical analysis

Data were plotted and analyzed using GraphPad Prism 8.0.1. software. Statistical significance was determined using the unpaired two‐tailed Student's *t*‐test or one‐way analysis of variance followed by a post hoc Newman–Keuls test, and the survival rate between each two groups was statistically analyzed using the log‐rank (Mantel‐Cox) test, where appropriate, and considered significant at *p* < .05.

## AUTHOR CONTRIBUTIONS

Yuan Yuan, Shi‐Qing Li, and Guan Yang designed the study, analyzed most of the data, and drafted the manuscript. Shi‐Qing Li, Ya‐Ru Wang, and Zhong‐Liang Xie performed the majority of the experiments. Yan Wang, Zi‐Han Feng, Jian‐Hao Xu, Bing Yuan, and Yi‐Tong Zhang contributed to animal experiments. Jing‐Lin Wang and Yuan Yuan supervised the study and provided funds. All the authors approved the final version of the manuscript.

## CONFLICT OF INTEREST STATEMENT

The authors declare no conflict of interest.

## ETHICS STATEMENT

Animal study protocols were approved by the Ethics Committee of the Academy of Military Medical Sciences (ethics board approval number: IACUC‐DW ZX‐2020‐008).

## Supporting information



Supporting information

## Data Availability

All data are available in the main text or the supplementary materials. The raw sequencing data have been uploaded to the NCBI Sequence Read Archive (SRA, http://www.ncbi.nlm.nih.gov/sra) under accession number SUB13793496.
