# Supplementary material for: NLRP3 activation maintains intestinal epithelial barrier and reduces liver injury in alcoholic liver disease mice
Source: Clin Transl Med. 2024 Nov 28;14(12):e70099. doi: 10.1002/ctm2.70099 (PMC11602754; doi:10.1002/ctm2.70099)
Supplement: Supplementary file 1 — Supporting information [file CTM2-14-e70099-s001.docx]

**NLRP3 activation maintains** **intestinal epithelial barrier and reduces liver injury in ALD mice**

Shi-Qing Li ^1,3†^, Ya-Ru Wang^1,4†^, Zhong-Liang Xie^1†^, Yan Wang^1^, Zi-Han Feng^5^, Jian-Hao Xu^1^, Bing Yuan^1^, Yi-Tong Zhang^1^, Guan Yang^2*^, Jing-Lin Wang^1*^, Yuan Yuan^1*^

**Supporting Table S1. List of test kits used in this study.**

| **Test kits** | **Supplier** | **Reference** | **From** |
| --- | --- | --- | --- |
| AST | Biobase Biodustry (Shandong) Co. | 702138 | Shandong, China |
| ALT | Biobase Biodustry (Shandong) Co. | 70211 | Shandong, China |
| TG | Suzhou Grace Biotechnology Co. | G0910W | Suzhou, China |
| ZO-1 | Elabscience | E-EL-M1161c-96T | China |
| D-LA | Abcam | ab83429 | USA |
| PCT | Cusabio Technology Co. | CSB-E10371m | Wuhan, China |
| IL-1β | Cusabio Technology Co. | CSB-E08054m | Wuhan, China |
| IL-18 | Cusabio Technology Co. | CSB-E04609m | Wuhan, China |
| TNF-α | Cusabio Technology Co. | CSB-E04741m | Wuhan, China |
| IL-6 | Quanzhou jiubang Biotechnology Co. | ZC20188 | Quanzhou, China |
| IL-8 | Quanzhou jiubang Biotechnology Co. | ZC-20191 | Quanzhou, China |
| LPS | Quanzhou jiubang Biotechnology Co. | ZC20397 | Quanzhou, China |

**Supporting Table S2. List of primer sequence of defensin in this study.**

| **Gene** | **F/R** | **Sequence** |
| --- | --- | --- |
| *Defa2*（mα-defensin-2） | Forward | AGACACTTGTCCTCCTCTCT |
|  | Reverse | CTGCCTGCTCCTCAGTATTAG |
| *Defa4*（mα-defensin-4） | Forward | CCAGGGGAAGATGACCAGGCTG |
|  | Reverse | TGCAGCGACGATTTCTACAAAGGC |
| *Reg3b* | Forward | AATGGAGGTGGATGGGAATG |
|  | Reverse | CCACAGAAAGCACGGTCTAA |
| *Reg3g* | Forward | TTCTCAGGTGCAAGGTGAAG |
|  | Reverse | GGCATAGCAATAGGAGCCATAG |
| *Defb1*（mβ-defensin-1） | Forward | TAGTCTCTTCATCTGTGTTTTTGCATA |
|  | Reverse | TTCAGCGCCACTGAGCACAGAC |
| *Defb3*（mβ-defensin-3） | Forward | CCAGGCTGATCCTATCCAGG |
|  | Reverse | GTCCCATTCATGCGTTCTCT |

**Supporting Table S3. List of antibodies used Western Blot in this study**

| **Antibodies** | **Supplier** | **Species** | **Dilution** | **Reference** |
| --- | --- | --- | --- | --- |
| **Primary antibodies** | | | | |
| ZO-1 tight junction protein | Abcam | rabbit | 1:1000 | ab216880 |
| Claudin-1 | Abcam | rabbit | 1:1000 | Ab307692 |
| GADPH | Abcam | rabbit | 1:5000 | ab181602 |
| NLRP3/NALP3 | AdipoGen | mouse | 1:1500 | AG-20B-0014 |
| caspase-1(p20) | AdipoGen | mouse | 1:1000 | AG-20B-0042 |
| E-cadherin | Proteintech | rabbit | 1:1000 | 20874-1-AP |
| GSDMD | CST | rabbit | 1:1000 | 39754S |
| IL-1β | R&D | rabbit | 1:1000 | AF-401-NA |
| **Secondary antibodies** | | | | |
| Goat Anti-Rabbit IgG | Solarbio | goat | 1:5000 | SE134 |
| Goat Anti-Mouse IgG | Solarbio | rabbit | 1:5000 | SE131 |


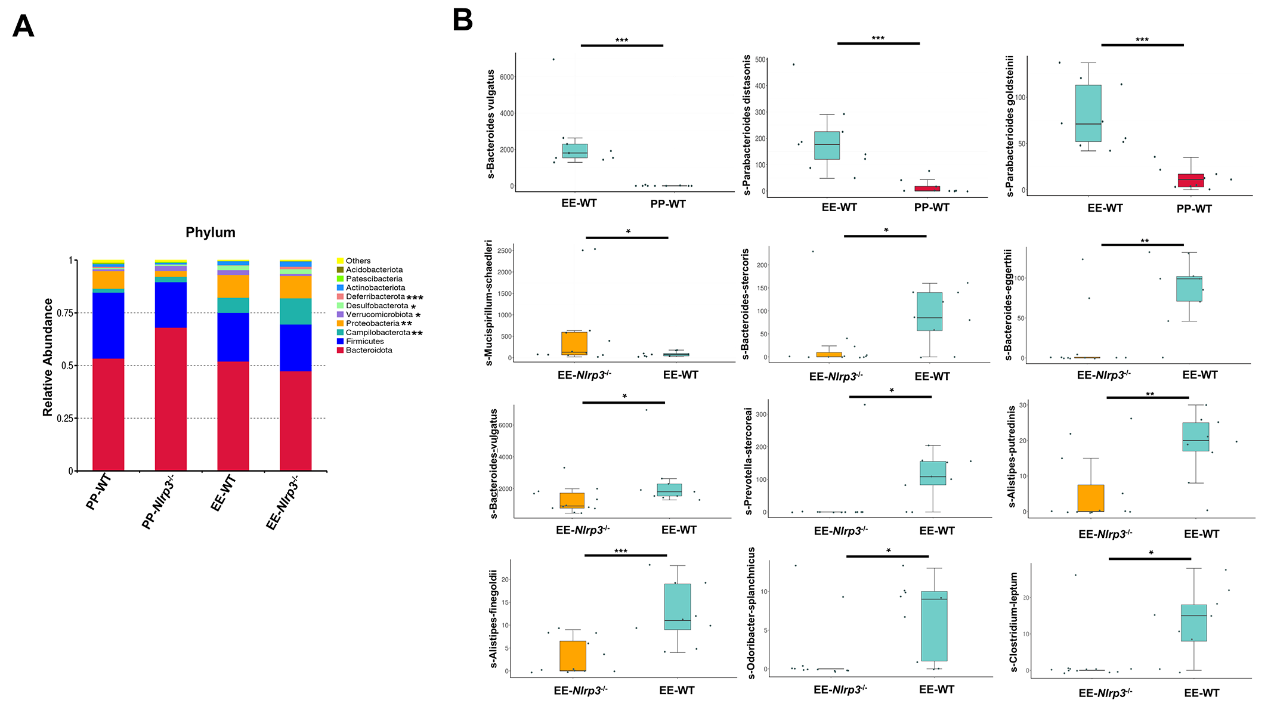


**Supplementary Figure. 1. Alcohol exacerbates Gut Dysbiosis in *Nlrp3*^-/-^ Mice.**

**(A)**Bacterial taxonomic profiling of intestinal bacteria from different groups at the phylum level. **(B)**Distribution box diagram of bacteria abundance with statistical difference at the species levels in WT mice from PP and EE group and WT and *Nlrp3*^-/-^ mice from the EE group using MetaStat analysis (box plots: median, 25th and 75th percentiles).

**
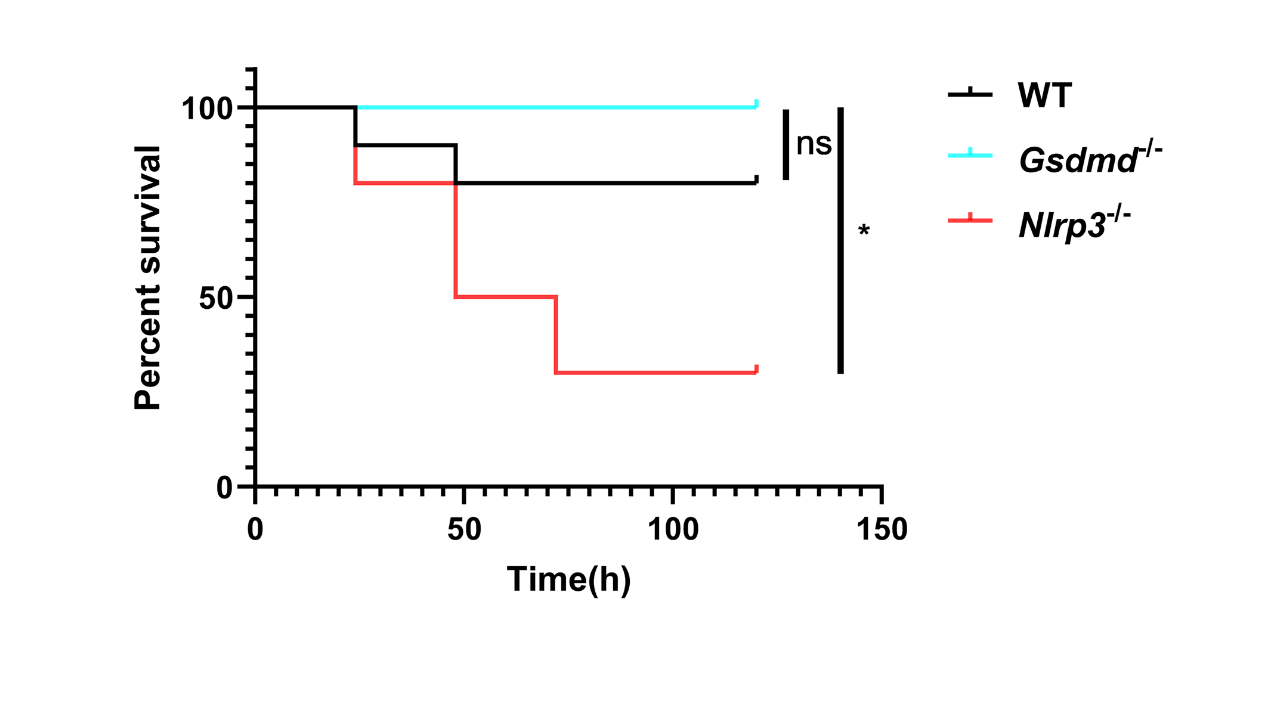
**

**Supplementary Figure. 2.** **NLRP3 protects from liver injury in ALD mice.**

Survival curve analysis of WT、*Gsdmd*^-/-^ and *Nlrp3*^-/-^ mice in EtOH group (i.e., alcohol feeding for 5 days after acute alcohol intragastric administration) (2 technical replicates, n=10). The survival rate between each two groups was statistically analyzed using the Log rank (Mantel Cox) test, ns *P* > 0.05, **P*<0.05.

**
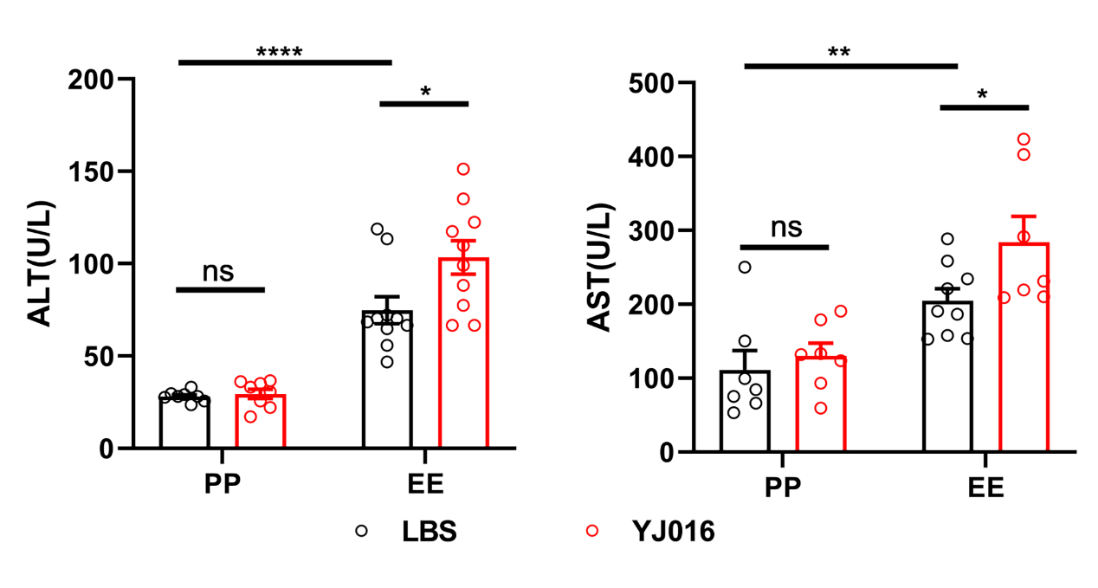
Supplementary Figure. 3.** **Infection of *V. vulnificus* in ALD mice exacerbates liver damage.**

Plasma AST and ALT were detected in healthy and ALD mice after foodborne infection with *V. vulnificus* YJ016.
